# Long term results of arthroscopic bankart repair for traumatic anterior shoulder instability

**DOI:** 10.1186/1749-799X-6-28

**Published:** 2011-06-14

**Authors:** Gerard WW Ee, Sedeek Mohamed, Andrew HC Tan

**Affiliations:** 1 Department of Orthopaedics, Singpapore General Hospital, Outram Road, Singapore 169608, Singapore

## Abstract

**Background:**

The arthroscopic method offers a less invasive technique of Bankart repair for traumatic anterior shoulder instability. We would like to report the 2 year clinical outcomes of bio-absorbable suture anchors used in traumatic anterior dislocations of the shoulder.

**Methods:**

Data from 79 shoulders in 74 patients were collected over 4 years (2004 - 2008). Each patient was followed-up over a period of 2 years. The patients underwent arthroscopic Bankart repair using bio-absorbable suture anchors for their shoulder instability. These surgeries were performed at a single institution by a single surgeon over the time period. The patients were assessed with two different outcome measurement tools. The University of California at Los Angeles (UCLA) shoulder rating scale and the Simple Shoulder Test (SST) score. The scores were calculated before surgery and at the 2-year follow-up. The recurrence rates, range of motion as well post-operative function and return to sporting activities were evaluated.

**Results:**

SST results from the 12 domains showed a significant improvement from a mean of 6.1 ± 3.1 to 11.1 ± 1.8 taken at the 2-year follow-up (p < 0.0001). Data from the UCLA scale showed a Pre and Post Operative Mean of 20.2 ± 5.0 and 32.4 ± 4.6 respectively (p < 0.0001). 34 had excellent post-operative scores, 35 had good scores, 1 had fair score and 3 had poor scores. 75% of the patients returned to sports while 7.6% developed a recurrence of shoulder dislocation or subluxation.

**Conclusion:**

Arthroscopic Bankart repair with the use of suture anchors is a reliable treatment method, with good clinical outcomes, excellent post-operative shoulder motion and low recurrence rates.

## Introduction

Recurrent shoulder dislocation or instability is common in young athletes. These injuries often occur during sports, preventing the individual from returning to these activities. The stability of the glenohumeral joint is maintained by the glenoid labrum. This labrum creates a socket-deepening effect hence preventing any shoulder dislocations.

An avulsion of this anterior inferior labrum from the glenoid rim was first described by Perthes and Bankart in the early twentieth century [1,2]. Since then, several open and arthroscopic techniques have been described to address anterior shoulder instability. These procedures address both capsuloligamentous laxity and labral pathologies via a variety of instruments, suture passages, knot-tying techniques and fixation devices. With the debate continuing regarding the indications for arthroscopic shoulder stabilization, recent studies have shown favorable outcomes with regards to the arthroscopic method [1,2]. Moreover, with continuing criticisms with regards to the wide dissection, loss of external rotation, and post-op pain associated with the open repair, the demand for arthroscopic surgery has increased over the last two decade.

However, despite advances in the understanding and techniques of arthroscopic surgery, failure rates have reported to be as high as 30%. As arthroscopic techniques have continued to evolve over the last decade, it is important to evaluate if these new techniques have resulted in an improved outcome.

The following study aims to report and evaluate the pre-operative evaluation, thorough diagnostic arthroscopic examination for concomitant pathology, surgical techniques and the postoperative therapy program for a successful outcome of arthroscopic Bankart repair with the use of bio-absorbable suture anchors for patients that were followed up for at least two years from the date of surgery.

## Methods

From 2004 to 2008, a total of 79 shoulders in 74 patients underwent arthroscopic Bankart repair for recurrent anterior glenohumeral instability by a single surgeon at our institution. Five patients had bilateral shoulders repaired. We hence conducted a retrospective analysis of a prospectively collected data after approval was sought for our study protocol from our hospital's ethics committee. 5 patients were lost to follow-up for UCLA analysis and 6 patients did not complete the SST questionnaire. Inclusion criteria for surgery included recurrent anterior glenohumeral subluxation or dislocation after an initial episode of traumatic anterior shoulder dislocation, a Bankart lesion confirmed by arthroscopic examination or ultrasound or Magnetic resonance imaging (MRI) and arthroscopic Bankart repair done using bio-absorbable suture anchors. The exclusion criteria were posterior instability, multidirectional instability, Hill-Sachs lesions more than 25% of the humeral head and bony Bankart lesion more than 25%. The degree of structural bony lesions was evaluated during arthroscopy, and patients demonstrating an engaging hill sacs or an inverted pear glenoid were taken to have significant bony loss [1]. All patients demonstrated a positive apprehension test as well as a load and shift test. All patients had pre-operative radiographs with an anterior-posterior, lateral, axillary and scapular-Y views taken. Magnetic resonance arthrograms were performed patients with equovical findings. The patients were included in the study after obtaining written, informed consent.

Two different outcome scoring measures were used to evaluate the effectiveness of the arthroscopic Bankart repair. The shoulder rating scale of University of California Los Angeles (UCLA) [1] and the simple shoulder test (SST) [1]. The SST consisted of a series of 12 yes-no questions, measuring pain and function of the shoulder through assessing the patient's ability to perform 12 simple tasks with the affected shoulder. The maximum total score was 12 points, with a higher score indicating better function. The UCLA was used to evaluate the patient's pain, function, forward flexion, strength and patient satisfaction. These five items are rated on ordinal scales of different lengths and scoring points. The maximum total score possible is 35, with a higher score indicating better shoulder function. We assigned a score of 34-35 points as excellent, 29-33 points as good, 21-28 as mild, and 20 or less as poor.

The UCLA and SST were chosen based on reproducibility, practicability, ease of use and ease of incorporation in clinical practice. We believe that they were the most responsive scoring systems and also most accurately reflect the outcomes of the surgery by assessing the tasks the patients are able to perform with the shoulder [1]. The UCLA has also shown to have a low inter-observer variability [1], while the SST has also been shown to satisfy the American Shoulder and Elbow Surgeons recommended attributes for a shoulder function assessment form [1]. Furthermore, these 2 outcome scores have also been used on numerous occasions in evaluating instability of the shoulder [1]. Data analysis comparing the pre-operative and post-operative UCLA scores were done using the Wilcoxon matched pairs test and data comparing the before and after surgery outcomes for the SST were done using the Unpaired T test. A value of p < 0.001 was taken as significant. All patients were followed up in clinic at 2 weeks, 1 month and then at 6 monthly intervals. All patients had a minimum of 2 years follow-up. Pre and post operative range of motion, function and return to sports were recorded. Treatment failure was regarded as recurrent shoulder dislocation, any sensation of subluxation, or instability preventing return to full activity or requiring a further stabilizing procedure.

### Surgical procedure

All operations were performed with the use of a standardised technique by the same surgeon. After induction of a general anaesthesia, the patient was placed in a beach chair position and a thorough examination under anaesthesia was performed to assess the magnitude and direction of instability. The shoulder was prepared and draped in a sterile manner, and the bony landmarks were marked carefully to maintain orientation throughout the procedure. A standard posterior viewing portal was established approximately 2 cm inferior and one cm medial to the acromial angle. Two anterior portals were established using outside-in technique with a spinal needle to establish the most appropriate placement of the cannulas. The anterosuperior portal was made in the rotator interval just inferior to the anterior edge of the acromion, and the anterior midglenoid portal was made just over the superior border of the subscapularis tendon. A small cannula was inserted into the anterosuperior portal, and a large-diameter threaded cannula was placed in the anterior midglenoid portal. Complete diagnostic arthroscopy was done through the posterior and anterior portals, with assessment of the glenoid labrum, capsule, rotator cuff and the humeral head for possible Hill-Sachs lesions. Rotator interval closure was not performed and any other tears of the glenoid labrum were repaired.

**Figure 1:**
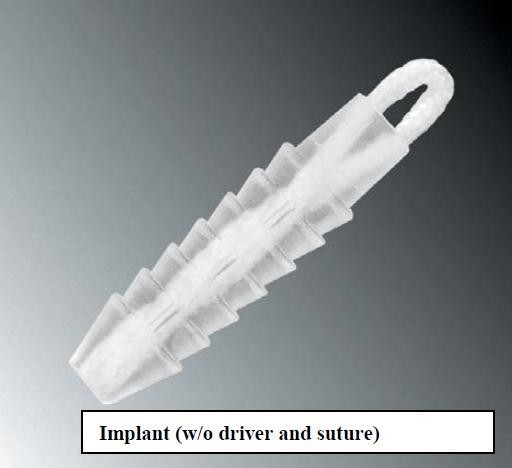
**Demonstrates the suture anchor used**.

The Bankart lesion was mobilised from the anterior glenoid surface using a periosteal elevator. The goal was to mobilise the labrum such that it could be shifted superiorly and laterally. The glenoid neck was lightly abraded using a rasper. All suture anchors used were from obtained from Arthrex. The Bio-suture Tak is a 3 mm diameter by 13 mm long bio-absorbable "push-in" anchor with a molded-in suture eyelet ideally suited for soft tissue attachment to bone in the shoulder joint where a small anchor profile with high pull-out strength is required. This suture anchor is molded from PLDLA poly (L-lactide-co-D, L-lactide), a non-crystalline, bio-absorbable copolymer. Figure 1 demonstrates the suture anchor used. The first anchor was placed at the 5.30 o'clock position, on the glenoid articular surface 3 mm from the articular edge. We believe this is essential in recreating the labral bumper, re-establishing the concavity compression effect and also tensioning the inferior glenohumeral ligament. The most inferior placement would ideally be placed at the 6 o'clock position however this often is not possible due to limitations in the placement angle. The suture anchor used requires drilling a pilot hole or using a punch to create the pilot hole prior to impaction of the implant to a countersunk position in the bone. A suture passer is then passed under the Bankart lesion. The suture strand of the suture anchor nearer the labrum was brought out through the anterosuperior portal, and in turn through the labrum in a retrograde fashion using the suture passer and retrieved from the midglenoid portal. This suture limb remained as the post during suture tying and this would ensure that the knot rest of the capsular side of the glenoid labrum and not on the articular side. This technique would effectively push the labrum up towards the glenoid socket, restoring labral height [1] and thereby recreating the labral bumper. Lazarus et al showed in a cadaveric study that by reducing the labral height by 80%, the resultant stability of the joint was decrease by 60% and that restoring of the labral height was paramount in restoring stability of the glenohumeral joint [1]. Hence our goal through the above techniques described through anatomical restoration of labral complex we hope to restoring tension in the anterior inferior glenohumeral ligament and achieve stability of the glenohumeral joint.

The second and third suture anchors were done at the 4.30 and 3.30 clock positions in the same manner. The sutures were tied using the Tennessee slider knot, which is easy to tie, has a low profile and possesses good holding strength [1]. When there was evidence of anteroinferior capsular laxity, the suture passer would be passed through the perilabral capsule one cm anterior and one cm inferior to the Bankart lesion to plicate the redundant capsule. This laxity is assessed by the ability to pass the arthroscope between the humeral head and the glenoid at the level anterior band of the inferior glenohumeral ligament. This drive-through sign is considered to be diagnostic of shoulder laxity or instability [1].

Postoperatively, the patients were placed in a sling for six weeks. They were allowed to do pendular motion exercises for the first three weeks, followed by elevating the elbow to shoulder level (forward active flexion to 90°) from the third to the sixth week. They were also taught to do isometric rotator cuff exercises during these six weeks. Full shoulder mobilisation was allowed after six weeks. Sport activities were allowed at three months and contact sports at four months.

### Biostatistics

Table 1 demonstrates the biostatistics of the patients in this study. There were no complications with regards to the arthroscopic technique. No bleeding, infection, compartment syndrome or neurological compromise were observed post-operatively. The most common associated injury was a Hill-Sach's lesion. This occurs as the posterior aspect of the humeral head impacts against the anterior glenoid, when the shoulder is discloated anteriorly.

**Table 1:** Bio-statistics of the patients who underwent Arthroscopic Bankart repair

Average Age (range) (*years*)	24.85 (13-44)
Gender	
Male	74
Female	1
Number of shoulders*	79 (5)
Mean number of dislocations before surgery (range)	11.17 (1-100)
Mean duration of operative time (range)	64.56 (35-145)
Mean pre-operative range of external rotation (range)	79.60 (60-90)
Mean post-operative range of external rotation (range)	81.39 (60-90)
Mean number of suture anchors (range)	2.87 (2-3)
Operative finding (Number of shoulders)	
Bankart lesion	79
Hill-Sachs lesion (mild grade)	10
Chondrolabral lesion	1
Bony Bankart lesion > 25% SLAP lesion	0 2
Lax anteroinferior capsule	11
(required capsular plication )	1
Fraying biceps tendon associated with severely-inflamed capsule	

## Results

The Simple Shoulder test (SST) showed a total of 73 responses out of the 79 shoulders that were operated on. The SST showed statistically significant improvement (P < 0.0001) from the pre-operative scores from a mean and standard deviation (SD) of 6.06 ± 3.12 with a range from 0 to 8 to a mean and SD of 11.08 ± 1.78 and a range from 4 to 12.

Table 2 demonstrates the scores from The UCLA evaluated the patient's pain, function, active forward flexion, strength of forward flexion and satisfaction of the patient. Total UCLA score showed an improvement from a mean and SD of 20.21 ± 4.98 before surgery to 32.44 ± 4.60 post surgery, with 69 shoulders achieving excellent or good scores (94.5%), 1 having a fair score (1.5%), and 3 having poor scores (4.1%). All patients demonstrated good range of motion with a mean and SD external rotation of 81.39 ± 8.12 degrees.

**Table 2:** UCLA outcome scores in patients after an arthroscopic Bankart repair with suture anchors

	Mean and SD before surgery (n = 73)	Mean and SD after surgery (n = 73)	P Value (Unpaired T)
Pain	5.84 ± 2.33	8.79 ± 1.62	< 0.0001
Function	5.74 ± 2.54	9.18 ± 1.69	< 0.0001
Active Forward Flexion	4.44 ± 0.91	4.95 ± 0.23	< 0.0001
Strength of forward flexion	4.05 ± 1.10	4.79 ± 0.55	< 0.0001
Satisfaction of patient	0	4.73 ± 1.15	< 0.0001
Total	20.21 ± 4.98	32.44 ± 4.60	< 0.0001
	Pre-operative		Post-operative
Number of shoulders who scored poor	36		3
Number of shoulders who scored fair	32		1
Number of shoulders who scored good	5		35
Number of shoulders who scored excellent	0		34

A total of 6 shoulders in 5 patients had a recurrence of shoulder instability. Of the 6, 4 of the recurrence of dislocation were due to sporting activities, while the causes of dislocation of 2 shoulders were unknown. 75% of the patients returned to previous sporting activities, while the remainder felt they could not return because they were afraid of a recurrence. All of the patients apart from those who developed a recurrence demonstrated a negative load and shift as well as a negative anterior apprehension test on post-operative clinical examination. Patients were also asked to rate the feeling of stability of their shoulder pre and post operation on a scale of 0 to 10, with 10 being the most unstable. Mean shoulder instability score was 7.33 before surgery and 1.89 after surgery.

No correlation could be established between the age, gender, frequency of dislocation, duration from first dislocation to surgery and the rate of recurrence. Although Voos and his colleagues found associated ligamentous laxity and age under 25 to be risk factors for recurrence, these factors could not be established in our study [1].

## Discussion

Historically, arthroscopic repair for the treatment of the Bankart lesion has been less satisfactory than the open technique [4]. However, many of these arthroscopic techniques described were using transglenoid sutures or bio-absorbable tacks [1]. In last few years, newer techniques involving suture anchor fixation and capsular pilacation have started to evolve, with promising results. These suture anchors have increasingly been use in laberal repair and capsulolabral reconstruction [1]. Our study has shown that patients undergoing arthroscopic repair with these suture anchors have excellent clinical outcomes and similar recurrence rates as compared to open surgery.

Suture anchors are low-profile fixation devices that minimize articular surface damage of the humeral head, offering anatomic reconstruction of the glenoid labrum as well as the glenohumeral ligament complex. These suture anchors may be inserted either open or arthroscopically, with the aim of re-attaching the anterior inferior labrum along with the ligaments to the glenoid labrum. Knots are placed on the capsular side of the Bankart lesion, recreating the socket-deepening bumper effect of the labrum and hence restoring the concavity-compression mechanism of the glenoid labrum on the humeral head [1]. Any redundant or lose capsule is also addressed during the same operation, allowing one to address any capsular laxity, restoring tension in the anterior-inferior glenohumeral ligament and stability to the glenohumeral joint.

The arthroscopic Bankart repair offers many advantages when compared to the open technique. It offers a minimally invasive approach with less surgical trauma and blood loss, with improvements in operating time, perioperative mobidity, narcotic use, hospital stay, time loss from work and decrease number of complications together with a lower cost of surgery [1]. We have also shown that post-operative range of motion is not sacrificed for the sake of stability, with a mean and standard deviation of 81.39 ± 8.12 degrees of external rotation. This allows the patients to return to sports or return to physically demanding jobs.

The introduction of bioabsorable suture anchors also simplifies any revision surgery, reducing concerns regarding infected implants [1] and anchor migration leading to articular cartilage damage [1]. During surgery, either two or three suture anchors are inserted, depending on the size of the Bankart lesion. Our results showed that patients who had only two suture anchors did not have a higher rate of recurrence. Patients with anteroinferior capsular laxity were treated accordingly by pinch tuck capsular placation as described earlier. Although some studies have shown that the presence of capsular laxity may affect the outcome of arthroscopic stabilization [1], while others have suggested that the elastic deformation of the glenohumeral ligament at the time of injury prevents the same degree of structural damage [1], we do not consider Bankart lesions associated with capsular laxity a contraindication to arthroscopic surgery. On the contrary, capsular placation can be done arthroscopically to address the issue of anteroinferior capsular laxity and this significantly augments the stability achieved with Bankart repair.

The majority of our patients were young physically active individuals, who engage of either vigorous sports or high demand jobs. Satisfactory range of motion, especially external rotation allows for performance during sports as well as proper functioning for activities during daily living. Several other studies published also reported a good range of motion after arthroscopic repair, often even better than repair with the open technique [1].

The recurrence rate in our study was 7.6%, which is similar to other published studies. Recurrence rates using the open technique ranged from 0-22% [1]. Warner et al initially published discouraging results with the arthroscopic techniques for contact sport athletics back in 1997 [1], however with modern arthroscopic techniques, extremely strong suture anchors and secure repair techniques allowing the patients to undergo extensive rehabilitation our study and other supporting studies have shown early return to competitive sporting activities [1,2].

## Conclusions

Arthroscopic Bankart repair with the use of suture anchors is a reliable treatment method, with good clinical outcomes, excellent post-operative shoulder motion and low recurrence rates.

## Competing interests

The authors declare that they have no competing interests.

## Authors' contributions

EWWG and SM were involved in all of the data collection, statistical analysis and interpretation as well as drafting of the final manuscript. TAHC was involved in editing the final manuscript and given the approval of the final version to be published. All authors have read and approved the final manuscript.

## References

[B1] Perthes G Über Operationen bei habitueller Schulterluxation Dtsch Z Chir 1906 56 149 51

[B2] Bankart ASB Recurrent or habitual dislocation of the shoulder BMJ 1920 1 1132 3 10.1136/bmj.2.3285.1132 PMC2317614 20771383

[B3] Sperling JW Smith AM Cofield RH Barnes S Patient perceptions of open and arthroscopic shoulder surgery Arthroscopy 2007 23 361 6 10.1016/j.arthro.2006.12.006 17418327

[B4] Fabbriciani C Milano C Demontis A Arthroscopic versus open treatment of Bankart lesion of the shoulder: A prospective randomized study Arthroscopy 2004 20 456 62 10.1016/j.arthro.2004.03.001 15122134

[B5] Lo Ian KY Parten Peter M Burkhart S Stephen M The inverted pear glenoid: an indicator of significant glenoid bone loss Arthroscopy The Journal of Arthroscopic and Related Surgery 2004 20 2 169 174 10.1016/j.arthro.2003.11.036 14760350

[B6] Ellman H Hanker G Bayer M Repair of rotator cuff. End-result study of factors influencing reconstruction J Bone Joint Surg Am 1986 68 113 44 3771595

[B7] Lippitt SB Harryman DT Masten FA Masten FA, Fu FH, Hawkins RJ A practical tool for evaluating function: The simple shoulder test The Shoulder: A Balance of Mobility and Stability 1993 Rosemont: American Academy of Orthopaedic Surgeons 501 18

[B8] Godfrey J Hamman R Lowenstein S Briggs K Kocher M Reliability, validity, and responsiveness of the simple shoulder test: psychometric properties by age and injury type J Shoulder Singapore Med J 2008 49 9 681 Elbow Surg 2007; 16:260-7 10.1016/j.jse.2006.07.003 17188906

[B9] Lam JJ Ip FK Wu WC Shoulder assessment systems: a comparison of three different methods Hong Kong J Med Sports 2000 XI

[B10] Richards RR An K-N Bigliani LU A standardized method for the assessment of shoulder function J Shoulder Elbow Sur 1994 3 347 352 10.1016/S1058-2746(09)80019-0 22958838

[B11] Sisto DJ Revision of failed arthroscopic Bankart repair Am J Sports Med 2007 35 537 41 10.1177/0363546506296520 17244898

[B12] Slabaugh MA Friel NA Wang VM Cole BJ Restoring the labral height for treatment of Bankart lesions: a comparison of suture anchor constructs Arthroscopy 2010 26 5 587 91 10.1016/j.arthro.2009.09.010 20434654 PMC3873634

[B13] Lazarus MD Sidles JA Harryman DT II Matsen FA III Effect of a chondral-labral defect on glenoid concavity and glenohumeral stability. A cadaveric model J Bone Joint Surg Am 1996 78 94 102 8550685 10.2106/00004623-199601000-00013

[B14] Baumgarten KM Wright RW Ease of tying arthroscopic knots J Shoulder Elbow Surg 2007 14 438 42 10.1016/j.jse.2006.10.013 17507243

[B15] McFarland EG Neira CA Gutierrez MI Cosgarea AJ Magee M Clinical significance of the arthroscopic drive-through sign in shoulder surgery Arthroscopy 2001 17 1 38 43 10.1053/jars.2001.19967 11154365

[B16] Voos JE Livermore RW Feeley BT Altchek DW Williams RJ Warren RF Cordasco FA Allen AA Prospective evaluation of arthroscopic bankart repairs for anterior instability Am J Sports Med 2010 38 2 302 7 Epub 2009 Dec 22 10.1177/0363546509348049 20028847

[B17] Freedman Kevin B Smith Adam P Romeo Anthony A Cole Brian J Bach Bernard R Jr Open Bankart Repair Versus Arthroscopic Repair With Transglenoid Sutures or Bioabsorbable Tacks for Recurrent Anterior Instability of the Shoulder Am J Sports Med 2004 32 6 1520 1527 10.1177/0363546504265188 15310581

[B18] Rudzki JR Purcell Derek B Wright Rick W Options for glenoid labral suture anchor fixation Operative techniques in sports medicine 2004 12 4 225 231

[B19] Lippitt S Matsen F Mechanisms of Glenohumeral Joint Stability Clin Orthop 1993 291 20 8504601

[B20] Conrad Wang Navid Ghalambor Bertram Zarins Jon JP Warner MDa Arthroscopic Versus Open Bankart Repair: Analysis of Patient Subjective Outcome and Cost Arthroscopy The Journal of Arthroscopic & Related Surgery 2005 21 10 1219 1222 16226650 10.1016/j.arthro.2005.07.004

[B21] Ticker JB Lippe RJ Barkin DE Infected suture anchors in the shoulder Arthroscopy 1996 12 613 5 10.1016/S0749-8063(96)90202-9 8902137

[B22] Berg EE Oglesby JW Loosening of a biodegradable shoulder staple J Shoulder Elbow Surg 1996 5 76 8 10.1016/S1058-2746(96)80035-8 8919447

[B23] Neri BR Tuckman DV Bravman JT Arthroscopic revision of Bankart repair J Shoulder Elbow Surg 2007 16 419 24 10.1016/j.jse.2006.05.016 17531511

[B24] Habermeyer P Jung D Ebert T Treatment strategy in first traumatic anterior dislocation of the shoulder. Plea for a multi-stage concept of preventive initialmanagement Unfallchirurg 1998 101 328 41 10.1007/s001130050278 9629045

[B25] Fabbriciani C Milano C Demontis A Arthroscopic versus open treatment of Bankart lesion of the shoulder: A prospective randomized study Arthroscopy 2004 20 456 62 10.1016/j.arthro.2004.03.001 15122134

[B26] Cole BJ L'Insalata J Irrgang J Warner JJP Comparison of arthroscopic and open anterior shoulder stabilization: a two to six-year follow-up study J Bone Joint Surg Am 2000 82 1108 1114 10954100 10.2106/00004623-200008000-00007

[B27] Warner JJ Goitz RJ Irrgang JJ Groff YJ Arthroscopic assisted rotator cuff repair: patient selection and treatment outcome J Shoulder Elbow Surg 1997 6 463 72 10.1016/S1058-2746(97)70054-5 9356936

[B28] Amol Tambe Ravi Badge Lennard Funk Arthroscopic rotator cuff repair in elite rugby players Int J Shoulder Surg 2009 3 1 8 12 10.4103/0973-6042.50876 20616950 PMC2895299

[B29] Flurin PH Guillemette C Guillo S Traumatic rotator cuff tears in rugby players J Traumatol Sport 2007 24 203 6 10.1016/j.jts.2007.06.012

